# *Rickettsia asembonensis* Isolated from Four Human Cases with Acute Undifferentiated Febrile Illness in Peru

**DOI:** 10.3390/pathogens13060489

**Published:** 2024-06-08

**Authors:** Steev Loyola, Rosa Palacios-Salvatierra, Omar Cáceres-Rey, Allen L. Richards

**Affiliations:** 1 School of Medicine, Universidad Peruana Cayetano Heredia, Lima 15102, Peru; 2 Doctorado en Medicina Tropical, School of Medicine, Universidad de Cartagena, Cartagena de Indias 130014, Colombia; 3 Postgraduate Unit, School of Biological Sciences, Universidad Nacional Mayor de San Marcos, Lima 15081, Peru; 4 Doctorado en Ciencias Biológicas, School of Biological Sciences, Universidad Nacional Mayor de San Marcos, Lima 15081, Peru; 5 Biotechnology and Molecular Biology Laboratory, Centro Nacional de Salud Pública, Instituto Nacional de Salud, Lima 15072, Peru; 6 Department of Preventive Medicine and Biostatistics, Uniformed Services University of the Health Sciences, Bethesda, MD 20814, USA; allen.richards@comcast.net

**Keywords:** *Rickettsia asembonensis*, *Rickettsia*, Peru, rickettsial diseases, human infection

## Abstract

Rickettsioses, often underreported, pose public health challenges. *Rickettsia asembonensis* is a potential emerging pathogen that was previously detected in humans, animals, and a variety of arthropods. While its pathogenicity in humans remains unclear, it poses a potential public health threat. Here, we present an extended epidemiological, diagnostic, and genetic analysis of the information provided in a preliminary report on the investigation of rickettsiae in Peru. In particular, we report the detection of *R. asembonensis* in blood specimens collected from four human patients with an acute undifferentiated fever of a seven- to nine-day duration, all of whom tested negative for other vector-borne pathogens. Additionally, we describe the replicative capacity of the *R. asembonensis* isolates in cell cultures.

## 1. Introduction

Rickettsioses are neglected infectious diseases found across various locations throughout the Americas, resulting in its underreporting and under-recognition [1]. *Rickettsia asembonensis* is an emerging flea-borne agent, closely related to *Rickettsia felis* and other *R. felis*-like organisms, that belongs serologically to the spotted fever group of rickettsiae [SFGR] but genetically to the transitional group of rickettsiae [2]. *R. asembonensis* was first identified in Kenya in 2013, and since then, multiple studies have reported its global detection in a variety of arthropods collected from domestic, peri-domestic, and wildlife animals [2,3,4,5,6,7,8,9]. Although its pathogenicity and role as a human pathogen has not yet been elucidated, its detection in both human [10,11,12,13] and animal [14,15,16] specimens suggests a potential public health threat.

Rickettsial diseases have been extensively described across various regions of Peru through the identification of group-specific antibodies (typhus group rickettsiae [TGR] and SFGR) and/or rickettsial DNA in samples collected from human cases with febrile illness [13,17,18,19,20,21,22]. *R. asembonensis* has predominantly been detected in flea species of the genus *Ctenocephalides* collected from domestic or backyard animals belonging to individuals with an acute undifferentiated fever (AUF) or asymptomatic individuals in the Amazon basin and multiple other Peruvian urban and border areas [17,22,23]. However, it was not until 2018 that the detection of *R. asembonensis* in cultured samples from humans presenting with AUF in Peru was preliminarily reported [13]. Briefly, *R. asembonensis* was detected in four human leukocyte samples collected from the same number of cases in various Peruvian locations by using multiple cell lines, immunofluorescence assays (IFAs), and sequencing of a PCR-amplified short fragment of the *gltA* gene [13]. The lack of detailed methodology, epidemiological information, the use of complementary tests on primary samples and cultures, and the preliminary genetic analysis restricted to a set of four genetic references are constraints that have not been addressed to date. Here, our aim was to comprehensively provide and expand on the epidemiological information and genetic analysis that were preliminarily reported.

## 2. Materials and Methods

**Figure 1:**
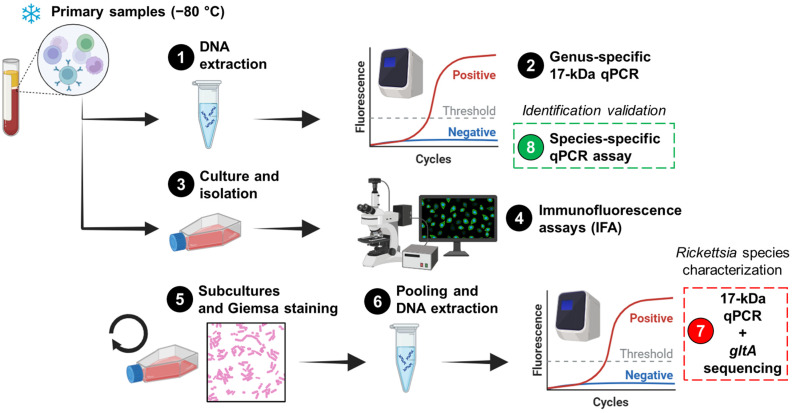
The workflow for the detection and characterization of *Rickettsia* in cultures and primary samples. The workflow comprises eight steps, starting with human blood leukocytes that were previously stored at −80 °C. The penultimate step (Step 7) involved the characterization of the agent in cultures, while the final step (Step 8) was performed to validate the results in primary samples. The figure was created in https://www.biorender.com/ (Accessed on 2 April 2024).

In 2009, a blood sample was obtained from an 11-year-old girl (case 1), and in 2010, three other blood samples were collected from a 23-year-old male (case 2), a 23-year-old female (case 3), and a 41-year-old female (case 4). All four cases described symptoms of AUF within the previous 7 to 9 days and were subsequently enrolled in the study “Pathogen investigation in human cases with acute undifferentiated febrile illness in Peru” at four distinct geographic locations in Peru (Table 1). The acute blood samples collected from each case were sent to the Peruvian National Institute of Health (PNIH) for the detection and characterization of multiple pathogens using advanced techniques not available at the enrollment sites. At PNIH, human serum/plasma and leukocytes were isolated from blood samples and were routinely screened for infections caused by various vector-borne viral (e.g., dengue virus serotypes 1–4) and bacterial (e.g., *Leptospira* spp.) pathogens, yielding negative results [13]. Since *Rickettsia* species detection was not included in the routine diagnostic procedures during the study period, the investigation of *Rickettsia* was conducted on human leukocytes (primary samples) preserved at −80 °C. Figure 1 depicts the workflow used in this study.

### 2.1. DNA Extraction

Genomic DNA from primary samples (Step 1; Figure 1) or pooled cell cultures (Step 6; Figure 1) was individually extracted and purified using the Blood & Cell Culture DNA Kit (Qiagen; Germantown, MD, USA) according to the manufacturer’s instructions.

### 2.2. Culture and Immunofluorescence Assay

Primary samples were inoculated into Vero E6 (African green monkey; ATCC CRL-1586), canine macrophage-like DH82 (ATCC CRL-3590), *Aedes albopictus* clone C6/36 (ATCC CRL-1660), murine macrophage-like J774A.1 (ATCC TIB-67), and/or human monocyte-like THP-1 (ATCC TIB-202) cells (Step 3; Figure 1) as described elsewhere [24]. Infected cells were maintained in Eagle’s Minimum Essential Medium (Merck; Darmstadt, Germany) supplemented with Earle′s salts, L-glutamine, non-essential amino acids, sodium pyruvate, and 5% fetal bovine serum (Merck; De Soto, KS, USA). Infections were conducted in 80% confluent monolayers within T-12.5 flasks, and inoculated flasks were incubated at 34 °C with 5% CO_2_ for 15 days. The presence of *Rickettsia* was assessed using an immunofluorescence assay (IFA) with *Rickettsia*-specific IgG antibodies (PanBio; Columbia, MD, USA) (Step 4; Figure 1). To increase the bacterial load and visualize at least 50% positive cells by IFA (Step 5; Figure 1), multiple subcultures were performed (Table 1). In these subcultures, Giemsa staining and light microscopy were used to monitor the infection (Step 5; Figure 1). However, it is important to highlight that the staining step was not critical for proceeding with the pooling of harvested subcultures or subsequent steps. When 20–50% IFA positivity was observed in each cell line, the cultures were harvested. Subsequently, 100 µL of each harvested culture was pooled, and genomic DNA was extracted (Step 6; Figure 1). IFA-negative cultures were excluded.

### 2.3. Molecular Assays

A genus-specific quantitative real-time PCR (qPCR) [25] and a species-specific qPCR for *R. asembonensis*, targeting the conserved 17 kDa surface antigen gene and the *ompB* gene [26], respectively, were performed as previously described using the 7500 Real-Time PCR System. The genus-specific 17 kDa qPCR assay was used on primary samples (Step 2; Figure 1) and pooled harvested subcultures (Step 7; Figure 1), while the species-specific qPCR was used only on primary samples (Step 8; Figure 1). Cycle threshold (Ct) values were recorded, and positive and negative controls were included in each run. Nested PCRs were performed to amplify regions of the conserved 17 kDa surface antigen and *gltA* genes, as well as the variable *ompB* gene, using previously described primers and conditions [27,28]. These PCRs were applied to primary samples to generate amplicons for subsequent sequencing.

### 2.4. Sequencing and Phylogenetic Analysis

PCR products were separated by electrophoresis on 2% agarose gels, purified, and then sequenced using the Big Dye terminator kit v3.1 (Thermo Fisher Scientific; Waltham, MA, USA) on the 3500XL genetic analyzer (Step 7; Figure 1). Sequences were analyzed and assembled in the BioEdit Sequence Alignment Editor v7.0.5.3, and consensus sequences were further analyzed using the nucleotide Basic Local Alignment Search Tool (BLAST; https://blast.ncbi.nlm.nih.gov/Blast.cgi; Accessed on 7 February 2024). Multiple *Rickettsia* species sequences were downloaded from the National Center for Biotechnology Information (NCBI) database (Table 2), and subsequently included in a phylogenetic analysis. The model was inferred by using the Maximum Likelihood method based on the Tamura 3-parameter model with a discrete Gamma distribution and 1000 bootstrap replicates. The phylogenetic analysis was conducted using Molecular Evolutionary Genetics Analysis (MEGA) X v10.2.6 software (https://www.megasoftware.net/; Accessed on 13 July 2023).

## 3. Results

All four primary samples were subjected to rickettsial DNA screening and displayed amplification signals by a genus-specific 17 kDa qPCR assay [25] (range of Ct values: 35.3 to 39.1). Water negative control samples were consistently negative. Based on the available sample volume, samples were cultured in at least two of the five cell lines described in Table 1 for the characterization of *Rickettsia* agents. All cultured samples were IFA-positive with the first attempt; and in the subcultures, coccobacillary microorganisms were identified by Giemsa staining. Subsequently, harvested cells were pooled and total genomic DNA was extracted [13].

**Figure 2:**
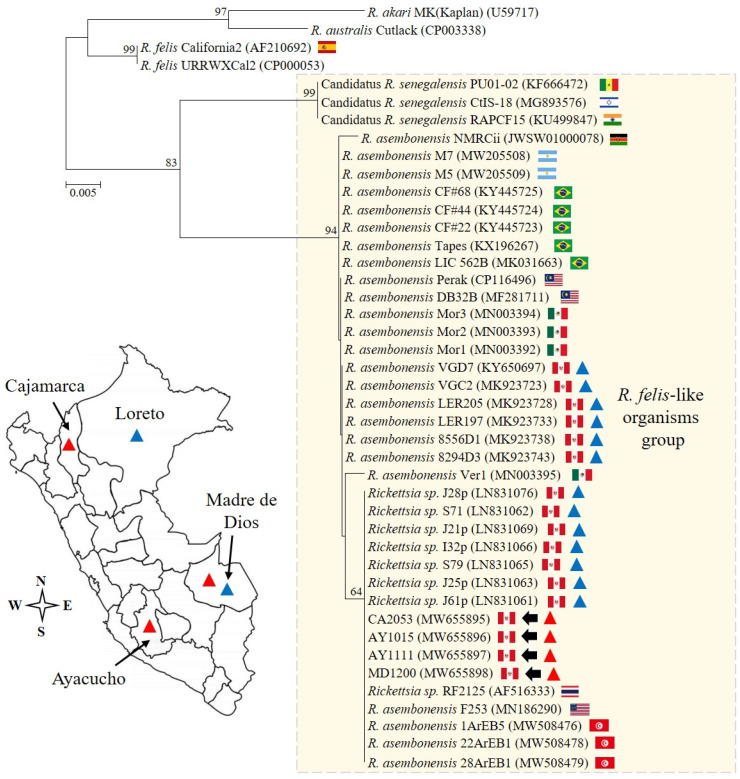
The phylogenetic analysis and detection sites. The analysis was performed including 28.8% (379/1314) of the full open reading frame of the conserved *gltA* gene from *Rickettsia asembonensis* strains and other *Rickettsia* species of the transitional group. The scale bar represents the number of substitutions per site, and the four sequences described in this study are marked by arrows. The genetic information reported for Peru is summarized in the genetic tree and map. Specifically, *R. asembonensis* detected in arthropods and humans is represented with blue and red triangles, respectively.

The *Rickettsia* species characterization was based on the following approach: (1) the detection of the 17 kDa antigen gene by qPCR [25], and (2) the sequencing and analysis of a segment of the *gltA* gene amplified by PCR [27]. From cultures, the Ct values derived from the 17 kDa qPCR assay were in the range of 25.3 to 29.1, and the sequencing of 379 nucleotides from the *gltA* gene confirmed the presence of *R. asembonensis* (GenBank accession numbers: MW655895–MW655898). Notably, all sequences described here were identical, and uninoculated control cultures and no-template controls tested negative by qPCR. A detailed comparative analysis with other *Rickettsia* sequences is summarized in Table 2, and the phylogenetic analysis is shown in Figure 2.

To validate our findings, a species-specific qPCR assay for *R. asembonensis* that targets a portion of the *ompB* gene was used on primary samples [26]. Amplification curves, although with high Ct values (36.3 and 38.9) suggestive of a low bacterial load, were observed in two of the four primary samples (Table 1). Importantly, the two primary samples that were negative for *ompB* gene amplification were positive for 17 kDa antigen gene amplification (samples from case 1 and case 4, Table 1). The detection limit for the genus-specific 17 kDa qPCR assay has been reported as three copies per reaction [25], whereas the detection limit for the species-specific qPCR has not been previously established [26]. These discrepant results may be attributed to a bacterial load below the detection limit for species-specific qPCR. Despite multiple attempts, we failed to generate *ompB* gene and 17 kDa antigen gene amplicons by standard nested PCRs from primary samples [28], as reported elsewhere for other genes [12]. The low bacterial load in primary samples likely limited our ability to generate PCR amplicons for sequencing.

## 4. Discussion

Our findings are consistent with those previously reported in two fundamental aspects. First, we successfully detected *R. asembonensis* in acute blood samples obtained from individuals exhibiting febrile illness and testing negative for pathogens other than *Rickettsia* [10,11,12]. Second, the methodology relying on partial sequencing of the *gltA* gene, as utilized here, has previously been used to confirm the presence of *R. asembonensis* in humans [10,11]. It is important to note that, compared to previous reports of *R. asembonensis* in humans [10,11,12], here, we describe not only its molecular detection but also its replicative capacity in multiple cell lines. In light of our findings, though it is not possible to conclusively assert that *R. asembonensis* was the causative agent of the patients’ diseases, we suggest further investigations to assess the pathogenicity of this novel agent. In subsequent investigations, the assessment of seroconversions/four-fold rise in titers through the examination of paired blood samples, a more complete culture, and genetic typing/detection (including more variable genes other than *ompB* and other conserved genes other than *gltA* and 17 kDa antigen) may contribute to a more robust determination of a rickettsial infection specifically occurring at the time of sample collections.

**Figure 3:**
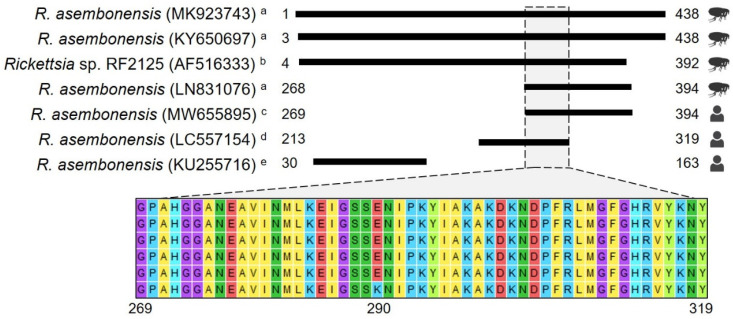
Amino acid alignment using *gltA* gene sequences. The figure depicts amino acid (aa) alignments using information deposited in GenBank for the full ORF of the *gltA* gene (438 aa). GenBank accession numbers are presented in parentheses, and numbers next to black boxes represent the aa positions. The first four sequences were obtained from arthropods collected in Peru ^a^ and Thailand ^b^, and the last three were obtained from human samples ^c–e^. The Thailand sequence was included because it is considered the potential first formal report of *R. asembonensis*. Since no differences were observed between sequences reported for arthropods among the studies published in Peru ^a^, MK923743 [29], LN831076 [23], and KY650697 [30] were selected to represent other sequences reported elsewhere. MW655895 is one of those reported in this study. LC557154 ^d^ is one of two sequences reported by Moonga et al. [11], and KU255716 ^e^ is the only sequence reported by Tay et al. [12].

Furthermore, it is important to highlight that our study encompasses a diagnostic and genetic analysis that supports the close genetic relationship between *R. asembonensis* documented here and strains previously identified in various geographic areas, vectors, and humans (Figure 2 and Figure 3). Noteworthy is that the *gltA* sequences reported in humans [10,11] and in vectors collected in Peru (Figure 3) are closely related. The sequence documented by Kho et al. [10] differs by only one amino acid (one base pair: guanine to adenine) compared to the sequences described herein, and the genetic regions compared between our sequences and those in vectors are identical. Understanding the biological implication of these findings was beyond the scope of this study; however, future research aimed at exploring the implications of our findings could greatly contribute to advancing our comprehension of the pathological role of *R. asembonensis*.

## 5. Conclusions

Our study adds to the epidemiological and genetic knowledge of *R. asembonensis* in humans and provides evidence that validates the presence of this agent in human samples.

## Figures and Tables

**Table 1:** Peruvian localities from which the cases were detected, and the laboratory results.

Case	Year	Province, Department (Coordinates)	Primary Sample *		Cell Lines **					Strain (GenBank Acc. No.)
			*17-kDa*	*ompB*	Vero	DH82	C6/36	J774A.1	THP-1	
1	2009	Santa Cruz, Cajamarca (6.6200° S, 78.9458° W)	38.2	Und.	3	N.T.	Neg.	N.T.	N.T.	CA2053 (MW655895)
2	2010	Vilcashuaman, Ayacucho (13.6541° S, 73.9521° W)	35.3	36.3	5	5	8	4	1	AY1015 (MW655896)
3	2010	Cangallo, Ayacucho (13.6291° S, 74.1438° W)	38.5	38.9	7	3	6	5	Neg.	AY1111 (MW655897)
4	2010	Tambopata, Madre de Dios (12.5825° S, 69.1933° W)	39.1	Und.	8	1	7	6	1	MD1200 (MW655898)

Note: * Cycle threshold (Ct) values are presented. Genomic DNA extracted from the primary samples was assessed by a qPCR assay that amplified a portion of the 17 kDa antigen gene conserved among *Rickettsia* species, and in a *R. asembonensis* species-specific qPCR assay that amplified a portion of the *ompB* gene. The absence of amplification signals is presented as Undetermined (Und.). ** Primary samples were cultured and subcultured in Vero E6 (African green monkey; ATCC CRL-1586), canine macrophage-like DH82 (ATCC CRL-3590), *Aedes albopictus* clone C6/36 (ATCC CRL-1660), murine macrophage-like J774A.1 (ATCC TIB-67), and/or human monocyte-like THP-1 (ATCC TIB-202) cells. Numbers represent the subcultures performed. N.T.: Not tested. Neg.: Negative results by IFA.

**Table 2:** Reference sequences of *Rickettsia* species and identity level against *R. asembonensis* isolated from human blood. Similarity values obtained by comparing the partial sequence of *gltA* from the Peruvian isolate CA2053 (accession number MW655895) and other sequences from multiple *Rickettsia* species and strains with BLAST (https://blast.ncbi.nlm.nih.gov/Blast.cgi; Accessed on 7 February 2024).

*Rickettsia* Species and Strain	GenBank No.	Country	Host	% Identity
*Rickettsia* sp. J28p	LN831076	Peru	*C. felis*	100.00
*Rickettsia* sp. S71	LN831062	Peru	*R. saguineus*	100.00
*Rickettsia* sp. J21p	LN831069	Peru	*C. felis*	100.00
*Rickettsia* sp. I32p	LN831066	Peru	*C. felis*	100.00
*Rickettsia* sp. S79	LN831065	Peru	*C. felis*	100.00
*Rickettsia* sp. J25p	LN831063	Peru	*C. felis*	100.00
*Rickettsia* sp. J61p	LN831061	Peru	*C. felis*	100.00
*Rickettsia* sp. RF2125	AF516333	Thailand	*C. canis*	100.00
*R. asembonensis* F253	MN186290	USA	*C. felis*	100.00
*R. asembonensis* 1ArEB5	MW508476	Tunisia	*A. erinacei*	100.00
*R. asembonensis* 22ArEB1	MW508478	Tunisia	*A. erinacei*	100.00
*R. asembonensis* 28ArEB1	MW508479	Tunisia	*A. erinacei*	100.00
*R. asembonensis* M5	MW205509	Argentina	*C. felis*	99.74
*R. asembonensis* M7	MW205508	Argentina	*C. felis*	99.74
*R. asembonensis* CF#68	KY445725	Brazil	*C. felis*	99.74
*R. asembonensis* CF#44	KY445724	Brazil	*C. felis*	99.74
*R. asembonensis* CF#22	KY445723	Brazil	*C. felis*	99.74
*R. asembonensis* Tapes	KX196267	Brazil	*R. sanguineus*	99.74
*R. asembonensis* DB32B	MF281711	Malaysia	*C. orientis/R. sanguineus*	99.74
*R. asembonensis* gltAMor3	MN003394	Mexico	*C. felis*	99.74
*R. asembonensis* gltAMor2	MN003393	Mexico	*C. felis*	99.74
*R. asembonensis* gltAMor1	MN003392	Mexico	*C. felis*	99.74
*R. asembonensis* 8294D3	MK923743	Peru	*C. canis*	99.74
*R. asembonensis* 8556D1	MK923738	Peru	*C. canis*	99.74
*R. asembonensis* LER197	MK923733	Peru	*C. canis*	99.74
*R. asembonensis* LER205	MK923728	Peru	*C. felis*	99.74
*R. asembonensis* VGC2	MK923723	Peru	*C. felis*	99.74
*R. asembonensis* VGD7	KY650697	Peru	*C. felis*	99.74
*R. asembonensis* Perak	CP116496	Malaysia	*C. orientis*	99.74
*R. asembonensis* LIC 562B	MK031663	Brazil	*C. felis*	99.74
*R. asembonensis* NMRCii	JWSW01000078	Kenya	*C. felis*	99.47
*R. asembonensis* gltAVer1	MN003395	Mexico	*C. felis*	99.47
*R. senegalensis* RAPCF15	KU499847	India	*C. felis*	96.05
*R. senegalensis* CtIS-18	MG893576	Israel	*C. felis*	96.05
R. senegalensis PU01-02	KF666472	Senegal	*C. felis*	96.05
*R. felis* URRWXCal2	CP000053	N.S.	N.S.	95.78
*R. felis* California 2	AF210692	Spain	Human	95.78
*R. australis* Cutlack	CP003338	N.S.	N.S.	94.74
*R. akari* MK(Kaplan)	U59717	N.S.	N.S.	94.21

Note: N.S.—Not specified.

## Data Availability

The original contributions presented in the study are included in the article; further inquiries can be directed to the corresponding author. Also, the data presented in this study are available in the National Center for Biotechnology Information at https://www.ncbi.nlm.nih.gov/nucleotide/ (Accessed on 14 June 2022), reference numbers MW655895.1; MW655896.1; MW655897.1; and MW655898.1.
